# Recent advances in understanding the relationship between long- and short-term weight change and fertility

**DOI:** 10.12688/f1000research.15278.1

**Published:** 2018-10-26

**Authors:** Audrey J. Gaskins

**Affiliations:** 1 Department of Nutrition, Harvard T.H. Chan School of Public Health, 665 Huntington Avenue, Boston, MA, 02115, USA; 2 Channing Division of Network Medicine, Brigham and Women’s Hospital and Harvard Medical School, 181 Longwood Avenue, Boston, MA, 02115, USA

**Keywords:** body weight, obesity, fertility, weight loss, weight gain, fecundity

## Abstract

The impact of excess body weight on fertility is well recognized among both women attempting to conceive spontaneously and those attempting to conceive with medical assistance. Although many leading societies of reproductive medicine have proposed weight loss as a means to counteract the negative consequences of obesity on fertility, there is limited research on this topic. In this review, we provide a brief overview of the recent advances in the literature focused on how long- and short-term weight change affects fertility among women. Overall, despite initial hope that weight loss may be beneficial for fertility, two large well-conducted randomized controlled trials have consistently shown that short-term weight loss among overweight and obese women undergoing infertility treatment does not improve a woman’s probability of live birth. The observational evidence among women attempting to conceive without medical assistance also suggests limited benefits of weight loss on fecundity or pregnancy loss. In contrast, substantial weight gain between consecutive pregnancies, in the year prior to pregnancy attempt, and throughout adulthood appears to be harmful for not only time to pregnancy but also pregnancy maintenance. Future research focused on efforts to prevent weight gain during adulthood is needed to better understand whether these types of interventions may have beneficial effects on fertility.

## Introduction

Obesity—defined as a body mass index (BMI) of at least 30 kg/m ^2^—is a major health problem globally because of its growing prevalence and substantial health implications related to chronic disease and mortality ^1^. Among reproductive-aged women, there is also mounting evidence that obesity is related to lower reproductive success, including higher risks of anovulation, irregular menses, infertility, miscarriage, and stillbirth ^2, 3^. In the US, the prevalence of obesity has steadily increased over the past decade such that now 37% of reproductive-aged women are considered obese, and the percentage is even higher among certain racial/ethnic minorities ^4^. This trend is not unique to the US or other developed countries, as similar phenomena have been observed worldwide ^5^.

Owing to the high prevalence of obesity among young women and the well-documented impacts of obesity on fertility, weight loss has been strongly promoted from the leading societies of reproductive medicine as one of the most effective means of increasing fertility in overweight or obese women ^6^. In some countries, obese women are even denied access to or funding for infertility treatment in the absence of substantial weight loss ^7, 8^. Despite these strong statements, the actual impact of weight change on fertility is not entirely clear. For example, the majority of evidence on which these recommendations are based involves studies comparing adverse reproductive outcomes in overweight or obese women compared with normal-weight women rather than directly evaluating the effects of weight loss.

In this review, we provide a brief overview of the recent advances in understanding the relationship between long- and short-term weight change and fertility among women. As fertility can be defined by a variety of endpoints, we have focused this review on the outcomes of time to pregnancy (TTP) and pregnancy loss among women attempting to conceive without medical assistance and clinical pregnancy and live birth following *in vitro* fertilization (IVF) among women undergoing assisted reproduction. There is increasing recognition that excess body weight in the male partner can also negatively affect couple fertility ^9, 10^, but, owing to a lack of studies on weight change and fertility outcomes in men, this was left out of the review.

## Short-term weight change and fertility

### Short-term weight change and assisted reproduction

Historically, studies on short-term weight loss interventions and fertility outcomes have focused on infertile obese women presenting for evaluation at fertility clinics. A systematic review of studies on this topic, published in 2014, nicely summarized the collective findings of the pioneering studies ^11^. Of the 11 articles included in the review, two were randomized controlled trials (RCTs), seven were cohort studies, and two were case reports. Eight of the studies implemented a dietary or lifestyle intervention program (or both) for weight loss, whereas three reported on a medical procedure for weight loss (for example, gastric bypass). The majority of studies (n = 9) included in the review were rated as having weak quality because of a less-than-rigorous study design, inappropriate control for confounders, high risk of selection bias, high rate of dropouts, or a combination of these. Moreover, the two RCTs on this topic, which were rated as having moderate quality, both had a total sample size of fewer than 50 women ^12, 13^. Despite these limitations, most studies found that weight loss prior to assisted reproduction was associated with improved chances of pregnancy and live birth among obese infertile women ^11^. Furthermore, other benefits regularly noted included regularization of menstrual cycles, a reduction in the number of infertility treatment cycles required, and a decrease in cancellation rates. Thus, the conclusion of this review was that the current clinical recommendation of advising overweight or obese women to lose weight prior to assisted reproduction was supported by the evidence, although larger prospective RCTs were needed.

Since this first review, many more articles, including an additional systematic review and meta-analysis that came out in 2017 ^14^, have been published on this topic. This updated review aggregated data from six RCTs on diet and exercise interventions aimed at weight loss and pregnancy outcomes among female infertility patients. Although this meta-analysis found higher pregnancy rates among the intervention groups compared with the control groups (relative risk [RR] = 1.59, 95% confidence interval [CI] 1.01, 2.50), there was not a significant difference in live-birth rates (RR = 1.54, 95% CI 0.93, 2.56). Interestingly, data from studies pre-dating the Lifestyle Trial by Mutsaerts *et al*., published in 2016, suggested that diet and exercise interventions were superior to standard care but the inclusion of the results from the Lifestyle Trial changed this conclusion ^15^. This is likely because the Lifestyle Trial was the biggest study to date and carried substantial weight in the meta-analysis. It also happened to be completely null.

The Lifestyle Trial was a large Dutch RCT that included 577 women randomly assigned to either a 6-month lifestyle intervention prior to 18 months of infertility treatment or prompt infertility treatment for 24 months ^15^. This seminal study found no differences in the proportion of women achieving a clinical pregnancy or live birth between the groups 2 years after randomization. In fact, women in the control group had a significantly shorter median TTP resulting in live birth (5.2 months) compared with the intervention group (8.8 months), likely because the former proceeded directly to infertility treatment. The only findings in support of weight loss were that women in the intervention group had more ongoing pregnancies that resulted from natural conceptions and the number of infertility treatment cycles was lower in the intervention group compared with the control. The benefit of weight loss on natural conceptions was also shown, in a follow-up article, to be driven specifically by anovulatory obese women at baseline, as the effect was much less pronounced among ovulatory women ^16^.

Although the findings of this influential RCT were mostly seen as discouraging, less than a year later, another large RCT of weight loss prior to IVF treatment among obese women was published with similar results ^17^. Though not as large as the original Dutch study, this RCT was the second largest, including 317 obese infertile women from Sweden, Denmark, and Iceland who were randomly assigned to an intervention consisting of 12 weeks of a low-calorie liquid diet followed by 2 to 5 weeks of weight stabilization or a control group with IVF only. Both groups of women were followed through the completion of one IVF cycle. By the end of follow-up, although women in the intervention group had more spontaneous pregnancies leading to live birth than did the control group, there were no significant differences in the proportion of women achieving clinical pregnancy or live birth across the two groups.

Prior to these trials, most scientists and clinicians believed that short-term weight loss would improve reproductive outcomes in obese women with infertility. The counterintuitive results from these two large, well-conducted RCTs, however, pretty conclusively showed that short-term weight loss for obese infertile women does not remedy the outcomes of IVF cycles. The RCTs also nicely highlight the importance of high-quality randomized trials, particularly in settings where common sense suggests that the answer is already known. An additional, large multi-site RCT investigating a similar question is ongoing in the US. However, until its results are published, the best evidence remains that short-term weight loss does not appear beneficial for most infertile obese women undergoing infertility treatment. The one exception to this rule consists of overweight and obese anovulatory women for whom weight loss increased the likelihood of spontaneous pregnancy prior to undergoing fertility treatment. In this subgroup, weight loss may negate the need for costly medical intervention to achieve pregnancy and any potential complications that may be associated with infertility treatment.

### Short-term weight change and natural conceptions

With regard to the benefits of short-term weight loss among overweight and obese women trying to conceive without medical assistance, the picture is less clear, as no RCTs have focused on this question. Although results from the two largest RCTs among women undergoing infertility treatment both suggested benefits of weight loss on achieving natural conception, it is important to remember that, in both trials, women in the control group received prompt infertility treatment and thus had less opportunity for unassisted pregnancy than did the intervention group.

In terms of observational evidence, the first studies on this topic focused on weight change between consecutive pregnancies and markers of fertility. In the first study, among 151,025 Swedish women, those with inter-pregnancy weight gains of 3 or more BMI units between their first two pregnancies had a 63% higher odds of stillbirth compared with those whose weight changed by less than 1 BMI unit ^18^. A later study among 218,389 American women found that normal-weight mothers who became overweight or obese, overweight mothers who became obese, and obese mothers who stayed obese across the two pregnancies under study had increased risk of stillbirth compared with normal-weight mothers who stayed at normal weight ^19^. With regard to effects on fecundity, among the 2,374 women who participated more than once in the Danish National Birth Cohort, those who were overweight or obese and lost or maintained weight between pregnancies had on average 5.50 (95% CI 1.35, 9.65) days shorter TTP for every 1 kg decrement in weight ^20^. Moreover, among women with a BMI of at least 18.5 kg/m ^2^, each 1 kg increment in weight gain was associated with 2.84 (95% CI 1.33, 4.35) days longer TTP.

The only other study on this topic did not focus on inter-pregnancy weight change but rather self-reported weight change in the year prior to pregnancy attempt. Among a preconception cohort of 629 women in the US, substantial weight loss in the past year (≥4 kg) was not associated with risk of pregnancy loss compared with maintaining a stable weight (RR = 0.99, 95% CI 0.56, 1.77) ^21^. This null association persisted when the analysis was restricted to overweight and obese women (RR = 1.27, 95% CI 0.51, 3.14). However, substantial weight gain in the previous year was associated with a marginally significant increased risk of pregnancy loss (RR = 1.41, 95% CI 0.98, 2.02 for at least 4 kg gain) relative to maintaining a constant weight. These findings, taken together with the conclusions from the studies evaluating inter-pregnancy weight change, suggest the potential importance of preventing short-term weight gain, rather than solely focusing on weight loss, among reproductive-aged women in order to increase fecundity and decrease risk of pregnancy loss.

## Long-term weight change and fertility

Owing to the challenges of randomly assigning women to long-term weight change interventions and the costs associated with continuing follow-up, the evidence on long-term weight change and fertility has come solely from observational cohort studies. Moreover, because of the daunting logistics of following a large group of women from late adolescence throughout their reproductive years, these studies have tended to define a woman’s long-term weight change on the basis of their recalled weight in late teenage years rather than measure this objectively or prospectively. The first study on this topic, published in 2010, found that among 1,651 Danish women planning pregnancy, women who gained 5 to 9 kg (fecundability ratio [FR] = 0.90, 95% CI 0.76, 1.07), 10 to 14 kg (FR = 0.86, 95% CI 0.70, 1.05), and 15 or more kg (FR = 0.72, 95% CI 0.59, 0.88) since age 17 had progressively lower fecundability (longer TTP) compared with women who remained weight-stable (±5 kg) ^22^. Women who reported a tendency to gain weight in their hips and thighs (but not those who gained weight in their stomach area) also had lower fecundability than did women with a tendency to gain weight equally all over. Among the small percentage of women who lost weight (4%), there was no difference in fecundability (FR = 1.05, 95% CI 0.73, 1.52).

These initial findings were followed by two separate TTP studies among women in the US. In the first, among 1,950 women in the Nurses’ Health Study 3 who were planning pregnancy, the median current duration of pregnancy attempt was non-significantly shorter (for example, −0.5 months) among the 127 women (7%) who lost at least 4 kg but significantly longer (for example, +1.4 months) among the 347 (18%) women who gained 20 or more kg since age 18 compared with women who maintained their weight (±4 kg) ^23^. Moreover, the detrimental effect of weight gain on TTP was shown to persist even among women who did not end up being classified as overweight or obese in adulthood. These significant findings for weight gain, however, were not confirmed in the most recent study among 2,062 women enrolled in the Pregnancy Study Online (PRESTO) cohort ^24^. Compared with women who were weight-stable (gained 0 to 9 lbs or 0 to 4 kg) since age 17, the 177 (14%) women who lost weight had slightly higher fecundability (FR = 1.11, 95% CI 0.93, 1.32) and the 236 women (19%) who gained 40 lbs or more (18 kg or more) had slightly lower fecundability (FR = 0.90, 95% CI 0.72, 1.12), but these differences were not statistically significant. Moreover, there were no significant differences in TTP according to where a woman tended to gain weight.

Finally, one observational cohort study evaluated long-term weight change and risk of pregnancy loss. Among 25,719 pregnancies reported by 17,027 women in the Nurses’ Health Study II, every 5 kg increase in weight since age 18 was associated with a 3% (95% CI 2, 4%) higher risk of pregnancy loss and women who gained the most weight (≥20 kg) had an 11% higher (95% CI 1, 23%) risk of pregnancy loss compared with women who maintained a stable weight (± 4 kg) since age 18 ^25^. Similar to before, the detrimental effect of weight gain on pregnancy loss was not entirely explained by current attained weight, as the association persisted even among women with a current BMI of less than 25 kg/m ^2^. Among women who lost weight since age 18 (≥4 kg), the risk of pregnancy loss was 20% lower (95% CI −29, −9%) compared with weight-stable women.

Among the handful of observational studies evaluating the association between long-term weight change and fertility, the findings regarding weight gain are relatively consistent: the more weight a woman puts on between late adolescence and prior to pregnancy, the longer it tends to take her to get pregnant and the higher her likelihood of pregnancy loss. Although current body weight appears to mediate some of the association between weight gain and decreased fertility, it does not completely explain it, suggesting alternative mechanisms. The other consistent finding across these studies was that very few women lost a substantial amount of weight since adolescence and, even among the women who did, it had little to no effect on TTP. Although weight loss might possibly have a beneficial effect on risk of pregnancy loss, this was evaluated in only one study. It is also worth noting that despite comparing pretty drastic long-term weight changes (for example, more than 20 kg or more than 40 lbs), the magnitudes of effects were actually quite modest: in the order of a month or two for TTP and a couple of percentage points for pregnancy loss. This suggests that there may be more effective interventions for increasing fertility in women beyond weight change efforts.

## Conclusions

Despite initial hope from small trials and observational studies, the current evidence, which includes two recently published, large RCTs, does not support promoting short-term weight loss as a means to increase fertility among overweight and obese women trying to conceive with medical assistance. Although there are no RCTs evaluating short-term weight loss and fertility among women conceiving without medical assistance, the observational evidence to date also suggests limited fertility benefits with weight loss. On the other hand, substantial weight gain between consecutive pregnancies, in the year prior to pregnancy attempt, and throughout adulthood appears to be consistently harmful for not only TTP but also pregnancy maintenance. This suggests that the obsessive focus on weight loss which has been the emphasis of clinical guidelines is perhaps misguided and efforts should be redirected to ones focused on the prevention of weight gain. Given the much higher prevalence of weight gain as compared with weight loss during young adulthood, any endeavor that can stymie weight gain during the reproductive years and in particular post-pregnancy will potentially have much larger public health implications for women as well.

## Abbreviations

BMI, body mass index; CI, confidence interval; FR, fecundability ratio; IVF, *in vitro* fertilization; RCT, randomized controlled trial; RR, relative risk; TTP, time to pregnancy
